# Apoptin Inhibits Glycolysis and Regulates Autophagy by Targeting Pyruvate Kinase M2 (PKM2) in Lung Cancer A549 Cells

**DOI:** 10.2174/1568009623666221025150239

**Published:** 2024-02-13

**Authors:** Gaojie Song, Chao Shang, Yilong Zhu, Zhiru Xiu, Yaru Li, Xia Yang, Chenchen Ge, Jicheng Han, Ningyi Jin, Yiquan Li, Xiao Li, Jinbo Fang

**Affiliations:** 1 Medical College, Jiujiang University, Jiujiang, 332000, China;; 2 Changchun Veterinary Research Institute, Chinese Academy of Agricultural Sciences, Changchun, 130117, China;; 3 Academician Workstation of Jilin Province, Changchun University of Chinese Medicine, Changchun, 130122, China

**Keywords:** Apoptin, PKM2, glycolysis, autophagy, lung cancer, energy metabolism

## Abstract

**Background:**

Pyruvate kinase M2 (PKM2) is a key enzyme in aerobic glycolysis and plays an important role in tumor energy metabolism and tumor growth. Ad-apoptin, a recombinant oncolytic adenovirus, can stably express apoptin in tumor cells and selectively causes cell death in tumor cells.

**Objective:**

The relationship between the anti-tumor function of apoptin, including apoptosis and autophagy activation, and the energy metabolism of tumor cells has not been clarified.

**Methods:**

In this study, we used the A549 lung cancer cell line to analyze the mechanism of PKM2 involvement in apoptin-mediated cell death in tumor cells. PKM2 expression in lung cancer cells was detected by Western blot and qRT-PCR. In the PKM2 knockdown and over-expression experiments, A549 lung cancer cells were treated with Ad-apoptin, and cell viability was determined by the CCK-8 assay and crystal violet staining. Glycolysis was investigated using glucose consumption and lactate production experiments. Moreover, the effects of Ad-apoptin on autophagy and apoptosis were analyzed by immunofluorescence using the Annexin v-mCherry staining and by western blot for c-PARP, p62, and LC3-II proteins. Immunoprecipitation analysis was used to investigate the interaction between apoptin and PKM2. In addition, following PKM2 knockdown and overexpression, the expression levels of p-AMPK, p-mTOR, p-ULK1, and p-4E-BP1 proteins in Ad-apoptin treated tumor cells were analyzed by western blot to investigate the mechanism of apoptin effect on the energy metabolism of tumor cells. The *in vivo* antitumor mechanism of apoptin was analyzed by xenograft tumor inhibition experiment in nude mice and immunohistochemistry of tumors’ tissue.

**Results:**

As a result, apoptin could target PKM2, inhibit glycolysis and cell proliferation in A549 cells, and promote autophagy and apoptosis in A549 cells by regulating the PKM2/AMPK/mTOR pathway.

**Conclusion:**

This study confirmed the necessary role of Ad-apoptin in the energy metabolism of A549 cells.

## 1 INTRODUCTION

Lung cancer is one of the most common malignant tumors in the world. Non-small cell lung cancer (NSCLC) accounts for approximately 80% of all lung cancers and causes 1.7 million deaths worldwide and every year. Although surgical resection is the traditional strategy of treating tumors with destitute long-term adequacy, treatment with chemotherapeutic drugs can lead to tumor resistance. Finding drugs that more specifically target cancer cellshas always been the focus of anticancer research.

The main characteristic of cancer cells is metabolic reprogramming. In the case of oxygen supply, most cancer cells rely on glycolysis to provide the energy they need [1, 2]. This aerobic glycolysis is also known as the Warburg effect, where pyruvate kinase M2 (PKM2) plays a decisive role [3]. PKM2 is one of the isoenzymes of pyruvate kinase (PK), which catalyzes the final reaction of glycolysis, in which the high-energy phosphate group is transferred from phosphoenolpyruvate (PEP) to ADP to form pyruvate and produce ATP [4]. The mammalian pyruvate kinase has four isomers: PKM1, PKM2, PKL, and PKR. PKM2 is highly expressed in cancer cells, such as gastric cancer [5, 6], hepatocellular carcinoma [7,8], breast cancer [9], cervical cancer [10], and lung cancer [11]. Studies have found that PKM2 can promote cell proliferation, migration, and invasion [12, 13]. PKM2 overexpression accelerates cancer cells' carcinogenic growth and autophagic inhibition [14]. In addition, Chu *et al.* reported that PKM2 knockdown can induce apoptosis and autophagy [15]. These findings indicate that PKM2 plays an important role in tumors’ metabolic and non-metabolic processes of tumor, and therefore, PKM2 may be a promising target for cancer therapy.

Apoptin is a small molecular weight apoptosis-inducing protein extracted from chicken anemia virus and can selectively kill a variety of human tumors cells without toxicity to normal human cells [16, 17]. Apoptin can induce tumor cell-specific apoptosis independently of p53, and therefore, can kill tumor cells even if the p53 gene is mutated or inactivated. Studies have shown that apoptin has a wide range of antitumor properties and is considered a potential biological antitumor protein [18-20].

Apoptosis is a highly regulated process of cell death; however, apoptosis is not the only mechanism determining cell fate. Autophagy, known as type II programmed death, may also be involved in regulating cell death. It is an important player in maintaining homeostasis and occurs in cells under nutritional deficiency, reactive oxygen species, hypoxia, drug stimulation, and endoplasmic reticulum stress. However, excessive cell damage can activate autophagy and turn it into cell death, and therefore, apoptosis and autophagy may co-regulate a broad range of cell death events. In a previous study, we constructed a viral vector expressing apoptin, Ad-apoptin, using the human adenovirus type 5. Compared with conventional transfection reagents, this delivery system has effectively expressed apoptotic proteins in cells [21]. Studies have shown that the expression of apoptin by oncolytic adenoviruses can induce tumor cell apoptosis through the intrinsic (or mitochondrial) pathway and regulate autophagy [21-23]. However, the relationship between apoptin-induced tumor cell death and energy metabolism has not been elucidated. Here, we utilized the same adenovirus vector system to evaluate the effects of apoptin on energy metabolism, apoptosis, and autophagy in PKM2 overexpressing or knockdown lung cancer cells *in vitro* and *in vivo* and confirmed the anti-tumoral role of apoptin and its potential application in cancer treatment.

## 2 MATERIALS AND METHODS

### 2.1 Cells and Viruses

The cell lines A549, BEAS-2B, NCI-H23, NCI-226, NCI-446, and A549/paclitaxel were purchased from the Cell Bank of the Shanghai Institute for Biological Sciences (Shanghai, China). The recombinant adenoviruses Ad-apoptin were constructed and preserved in the Chinese Academy of Agricultural Sciences (Changchun, China).

### 2.2 Reagents and Antibodies

The protease inhibitor cocktail (cat. NO. K1007) and the phosphatase inhibitor cocktail (cat. NO. K1015) were purchased from APExBIO Technology LLC (Shanghai, China). Primary antibodies for AMPK Phospho-AMPKα (cat. NO.2535), Phospho-mTOR (cat. NO. 5536), mTOR(cat. NO. 2983), Bcl-2 (cat. NO. 15087), Phospho-4E-BP1 (cat. NO. 2855), 4E-BP1 (cat. NO. 9644), GLUT-1 (cat. NO. 12939), Phospho-PKM2 (cat. NO. 3827), PKM2 (cat. NO. 4053), HK1 (cat. NO. 2024), HK2 (cat. NO. 2867), LDHA (cat. NO. 3582), PKM1 (cat. NO. 3190), PARP (cat. NO. 9532), Beclin-1 (cat. NO. 3495), Phospho-ULK1 (cat. NO. 14202), ULK1 (cat. NO. 8054), LC3A/B (cat. NO. 12741) and β-Actin (cat. NO. 4970) were purchased from CST, Inc. (Danvers, MA, USA).

### 2.3 Crystal Violet Staining

A549 cells were passaged at 2×10^5^ cells/well into 12-well plates and cultured overnight. Subsequently, the culture medium was discarded, and 500 ul of RPMI-1640 medium without FBS was added. Meanwhile, Ad-apoptin or transfection reagent was added as a treatment for 36 h. The cell culture medium was discarded, washed with 1×PBS, fixed with paraformaldehyde, and stained with crystal violet. Finally, after washing with 1×PBS, the plate was left to dry at room temperature and photographed.

### 2.4 siRNA and PKM2 Plasmids Transient Transfections

Small interfering RNAs (siRNAs) for *PKM2* were designed and synthesized by Ribobio (Guangzhou, China). The sequence of the siRNA that specifically targeted PKM2 mRNA was 5'-CCAUAAUCGUCCUCACCAA-3'. PKM (NM_002654) pcDNA3.1-3xFLAG-C plasmid was designed and synthesized by FENGHUISHENGWU (Changsha, China).

For transfection, the cells were plated at a density of 5×10^5^ cells/well in 6-well plates and cultured in a serum-containing medium. Triplicate was performed for each group. When the cells were 80% confluent, siPKM2 duplexes were transfected into cells using riboFECT CP (Ribobio, C10511-05). Briefly, 1× riboFECT™ CP buffer was prepared by diluting riboFECT™ CP Buffer (10×) with sterile PBS at room temperature. After shaking, it was placed at room temperature to acclimate with the ambient temperature for later use. A total of 5 ul of 50 nM siPKM2 stock solution was diluted with 120 ul of 1× riboFEC™ CP Buffer reagent. A total of 12 ul riboFEC™ CP Reagent was added, and then the solution was incubated at room temperature for 20 min in a biological safety cabinet to prepare a transfection complex. The cell culture medium in the 6-well plate was discarded, and 1863 ul RPMI 1640 medium was added to each well, followed by the addition of the riboFEC™ CP transfection complex to the 6-well plate with gentle mixing. After transfection, the 6-well plate was placed in a cell culture incubator for 24 h, the cells were harvested for subsequent assays.

For the overexpression experiments, the cells were plated at a density of 5×10^5^ cells/well in 6-well plates and cultured in a serum-containing medium. Triplicate was performed for each group. When the cells reached 80% confluent, the PKM (NM_002654) pcDNA3.1-3xFLAG-C plasmid was transfected into cells using lipofectamine-3000, according to the instructions provided with the lipofectamine-3000 kit (Thermo Fisher, L3000015, USA). After 48 h incubation, the cells were harvested for subsequent assays.

### 2.5 Quantification of Glucose and Lactate Assays

As previously described, glucose uptake and lactate production by A549 cells in the different treatment groups were determined [23]. Briefly, A549 cells from different treatment groups were seeded in 6-well plates, and after their culture for 36 h, 10 ul of cell culture medium was collected from each treatment group for subsequent measurement, according to the manufacturer’s instructions.

### 2.6 CCK-8 Assay

CCK-8 assays were performed as previously described [23]. Briefly, A549 cells from different treatment groups were seeded in 96-well plates and cultured for 36 h. A total of 10 µl of CCK-8 solution was added to each well for 3 h. Finally, a multi-plate reader measured cell viability was measured at an absorbance at 450 nm.

### 2.7 Apoptosis Analysis

The cells in different groups were seeded in 6-well plates with cell-cover glass slides. After discarding the medium, the cells were fixed with paraformaldehyde for 20 minutes and then washed with PBS for 5 minutes. Annexin V-mcherry detection solution was added, followed by incubation in the dark for 15 min, and then photos were taken using a fluorescence microscope for analysis. each well was randomly photographed 3 times. Normal cells were not stained by Annexin V-mCherry, while Annexin V-mCherry stained apoptotic cells. The stronger the red fluorescence, the more obvious the apoptosis.

### 2.8 Immunofluorescence

A549 cells in the logarithmic growth phase were cultured in 12-well cell culture plates pre-placed with cell glass plates. After treatment, the medium was discarded and the cells were fixed with 4% paraformaldehyde for 30 min, washed with PBS for 5 min, and then blocked with Blocking Buffer (Beyotime, China) for 30 min. After that, the primary antibody was added and the plate was incubated overnight at 4 °C in the refrigerator and washed three times with PBS for 8 min each. Subsequently, the room-temperature secondary antibody was added and incubated for 1 hour. Finally, the nuclei were stained with a DAPI staining solution for 1 min and the cells were photographed under a fluorescence microscope. Each well was randomly photographed 3 times. The stronger the red fluorescence expression, the more the target protein expressed.

### 2.9 QRT-PCR

TRIzol reagent was used to extract RNA from the A549 cells. The cDNA was generated with random primers using the Reverse Transcription System (TakaRa). The primers were as follows: Forward: 5'-AGAAGGCTGGGCTCAT-TTG-3' and reverse:5'-AGGGGCCATCCACAGTCTTC-3' for GAPDH. Forward: 5'-GTCGAAGCCCCATAGTGAAG-3' and reverse: 5'-GTGAATCAATGTCCAGGCGG-3' for PKM2. The comparative threshold cycle (2-^ΔΔCt^) method was used to quantify the mRNA expression levels of these genes.

### 2.10 Western Blot Analysis

After the extraction of total protein from cells using the Minute™ Total Protein Extraction Kit, the protein concentration was determined with the BCA kit. Proteins were separated by 10% SDS-PAGE and transferred to PVDF membranes. The membranes were blocked with 5% BSA rapid blocking solution for 20 min, then PKM2, p-AMPK, p-ULK1 and other rabbit-derived monoclonal primary antibodies (1:1 000 dilutions) were added and incubated overnight at 4 °C. This step was followed by 4 times washes with TBST, each time 8 min. Finally, the goat anti-rabbit secondary antibody (diluted 1:3 000) was incubated at room temperature for 40 minutes, followed by 4 washes with TBST for 8 minutes each. ECL was added dropwise to develop color and expose the corresponding protein bands.

### 2.11 Co-immunoprecipitation

Cell proteins were extracted and kept on ice. A total of 1.0 μg IgG and 20 μl protein A/G beads were added to the protein supernatant of the negative control (IgG) group, and 20 μl protein A/G beads were directly added to the experimental group. The incubation was performed at 4°C for 1 h. After incubation, 2000g, the mixes were centrifuged at 2000g for 5 min at 4°C. The supernatant was collected, and 5 μl of antibody was added and incubated at 4°C overnight. Following the incubation, 80 μl protein A/G-beads were added, and the mixes were incubated at 4°C for 2 h, followed by centrifugation at 2000g for 5 min at 4°C. After centrifugation, the supernatant was carefully removed, and the immune system precipitate complexes were collected and washed 4 times with 1 ml of pre-cooled IP lysis buffer. This step was followed by centrifugation at 2000g for 5 min at 4°C each time, and the supernatant was discarded; then, 80 μl of 1×SDS loading buffer was added, boiled for 10 min, followed by centrifugation at 1000g for 5 min at 4°C to obtain the supernatant. Finally, 10 μl of supernatant was used for Western blotting.

### 2.12 Animals, Xenograft Model and Treatment

Female BALB/c nude mice were purchased from the Experimental Animal Center of the Academy of Military Medical Sciences of China. All experimental animal protocols were approved by the Institutional Animal Care and Use Committee (IACUC) of the Changchun Veterinary Research Institute, Chinese Academy of Agricultural Sciences (IACUC of AMMS-11-2021-043). All operations were performed under pentobarbital anesthesia, according to the standard animal procedure.

Female BALB/c nude mice (4-5 weeks, 20 ± 2 g) were subcutaneously inoculated with A549 cells (1×10^7^). After successful tumor-bearing, nude mice were randomly divided into a control group and an Ad-apoptin group (10 mice in each group). In the Ad-apoptin group, Ad-apoptin (1×10^9^ plaque forming units) in a volume of 100 µl in PBS was intratumorally injected every 3 days for a total of 10 treatments. The control group was intratumorally injected with PBS. The tumor volume was measured with a caliper every 5 days using the following formula: [(W2×L)/2; W, width; L, length; in cubic millimeters]. The tumors’ tissues were weighed and fixed with 4% paraformaldehyde for immunohistochemical detection.

### 2.13 Histopathological Examination

Tissue samples were embedded in paraffin, cut into 5 µm sections, and stained with H&E. The histopathological assessments were performed blindly by two board-certified pathologists. The tumor sections were deparaffinized and rehydrated by baking at 65°C for 1 h. After high-temperature antigen retrieval in sodium citrate buffer, the sections were blocked with goat serum for 30 min at room temperature and then incubated with anti-Ki-67, TUNEL, p-AMPK, PKM2, p-mTOR, p-4E-BP1 and LDHA rabbit polyclonal antibodies overnight at 4°C. The slides were removed and rewarmed at room temperature for 20 min. The sections were then incubated in the dark with goat anti-rabbit IgG for 1 h at room temperature and then stained using the DAB kit. Results’ assessment Criteria: Ki-67 staining was considered positive when the nuclei were stained yellow brown. The Ki-67 index was assessed at 400× magnification in 3 fields of view. The presence of brown granules in the cytoplasm and nucleus was the positive staining for the expression of PKM2, p-AMPK, p-mTOR, p-4E-BP1 and LDHA proteins in the detected tumor tissue. The darker the positive staining, the higher the protein expression.

### 2.14 Statistical Analysis

All data are presented as a mean ± SEM unless otherwise indicated and previously reported by Song *et al.* [23]. The statistical analysis was conducted using data from at least 3 independent experiments using SPSS 20.0 (SPSS Inc., Chicago, IL, USA). The results were statistically analyzed using the student’s t-test or one-way analysis of variance (ANOVA; *p* < 0.05 was used to indicate statistical significance).

## 3 RESULTS

### 3.1 PKM2 is Highly Expressed in Lung Cancer Cell Lines, and its High Expression in Cancer was Associated with a Low Overall Survival Rate

**Fig. (1):**
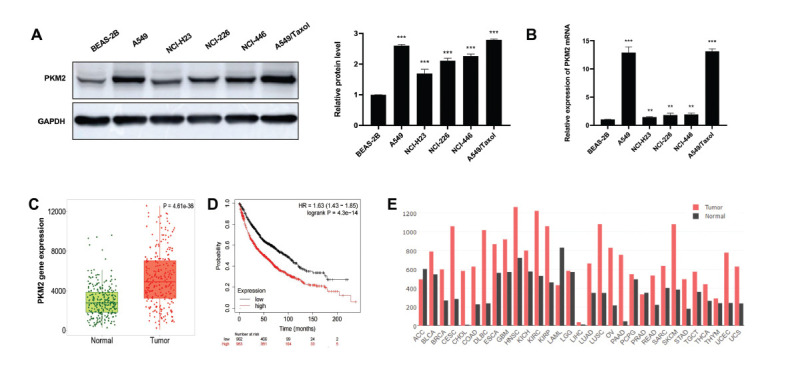
PKM2 high expression in cancer is associated with low overall survival rate. (**A**) PKM2 protein expression in 5 lung cancer cell lines and normal control cells. (**B**) PKM2 mRNA expression in 5 lung cancer cell lines and normal control cells was detected by qRT-PCR. The results are representative of three independent experiments. **p < 0.01, ***p < 0.001. (**C**) Comparison of Kaplan-Meier block diagram of PKM2 expression between lung cancer and normal lung tissues (normal lung specimens, n = 259; Tumor specimens, n = 259) (we performed Kaplan-Meier analysis using online portal (www.kmplot.com). (**D**) Kaplan-Meier survival curve (low expression group of PKM2, n = 962; PKM2 high expression group, n = 963) (we performed Kaplan-Meier analysis using online portal (www.kmplot.com). (**E**) Box plots of GEPIA (a web server for cancer and normal gene expression profiling and interactive analyses) show the expression of PKM2 in some tumor samples and paired normal tissues. The expression of PKM2 was higher in tumor samples.

Western blot and qRT-PCR analysis were used to compare the expression levels of PKM2 in different lung cancer cell lines (A549, NCI-H23, NCI-226, NCI-446 and A549/paclitaxel) and normal lung epithelial cells (BEAS-2B). PKM2 protein and mRNA levels in lung cancer cells were higher than those in normal human lung epithelial cells (BEAS-2B) (*p* < 0.001) (Figs. **1A** and **1B**), indicating that PKM2 is significantly expressed at high levels in lung cancer cell lines. We also analyzed the expression of PKM2 in the normal lung (n = 259) and lung cancer tissues (n = 259) using the Kaplan-Meier method (www.kmplot.com) and found that PKM2 expression in lung cancer tissues was 1.82 times higher than that in normal lung tissues (Figs. **1C** and **1D**). Using the Kaplan-Meier method, we also analyzed the relationship between the expression of PKM2 and the overall survival rate of cancer patients. PKM2 high expression was closely related to a low overall survival rate in lung cancer patients (Fig. **1D**). In addition, according to the data of the GEPIA database the PKM2 expression level in all tumor samples was higher than that in their paired normal tissues (Fig. **1E**). Our results are consistent with the above results and indicate that PKM2 is highly expressed in tumor tissues. The results also suggest that PKM2 may be a useful target for tumor therapy.

### 3.2 Apoptin Inhibits the Expression and Activity of PKM2 and Suppresses Aerobic Glycolysis in A549 Cells

**Fig. (2):**
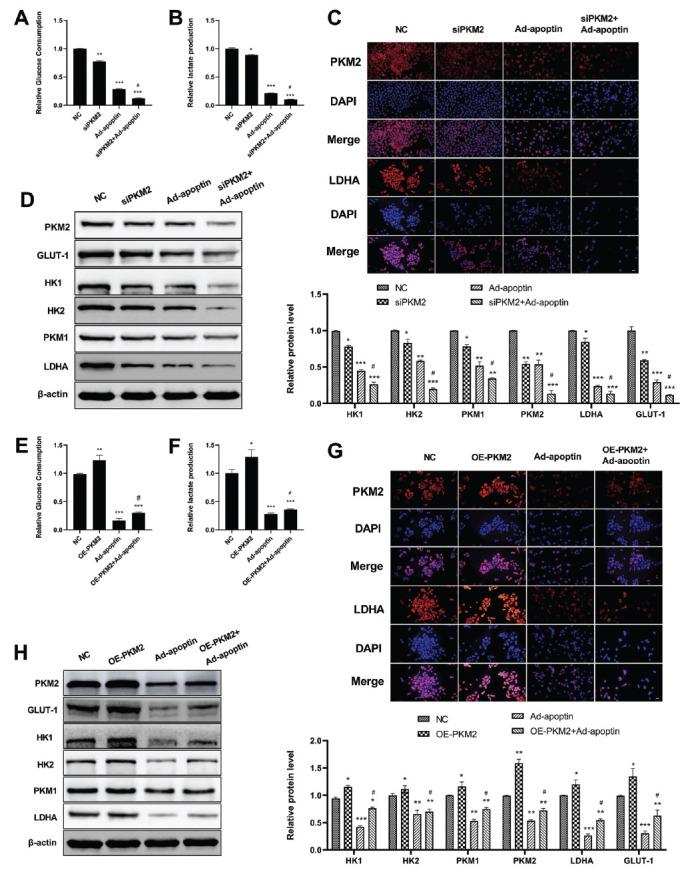
Ad-apoptin inhibits the expression and activity of PKM2 and suppresses aerobic glycolysis in A549 cells. Glucose consumption assay (**A** and **E**) and lactate production assay (**B** and **F**) in different treatment groups of A549 cells. (**D** and **H**) Western blot assessing the protein expression levels of PKM2, LDHA, HK1, HK2, PKM1, and GLUT-1 in A549 cells treated with NC, PKM2 siRNA, PKM2 over-expression or Ad-apoptin. (**C** and **G**) Immunofluorescence analysis of the expression of PKM2 and LDHA protein (red) in A549 cells. The nucleus was stained with DAPI (blue). Magnification: x200. scale bar = 50 μm. n = 3. *p < 0.05, **p < 0.01 and ***p < 0.001. *vs* the respective NC group; ^#^*p* < 0.05. siPKM2 + Ad-apoptin group or OE-PKM2 + Ad-apoptin group *vs* the respective Ad-apoptin group.

Aerobic glycolysis is a unique metabolic characteristic of cancer cells that is crucial for the proliferation of cancer cells. Glycolysis level is usually measured by glucose consumption and lactic acid production. The glucose uptake of A549 cells in the siPKM2 (PKM2 knockdown) group, Ad-apoptin (200 MOI/cell, the concentration was known by pre-experiment) group, and the Ad-apoptin + siPKM2 group was significantly decreased compared with that in the control group. This decrease was more pronounced in the Ad-apoptin + siPKM2 group (Fig. **2A**). The concentration of lactic acid in the culture medium of cells in the siPKM2 group, Ad-apoptin group, and Ad-apoptin + sipPKM2 group was significantly decreased compared with that in the control group. This decrease was more significant in the Ad-apoptin + siPKM2 group (Fig. **2B**). These results indicate that apoptin treatment reduces aerobic glycolysis in A549 cells, which is consistent with the results of the PKM2 knockdown experiments. Furthermore, we also investigated the protein (red fluorescence) expression levels of PKM2 and LDHA by immunofluorescence. Compared with the NC group, the fluorescence intensities of PKM2 and LDHA in the siPKM2 group, Ad-apoptin group and the Ad-apoptin + sipPKM2 group were significantly weaker (Fig. **2C**) and the PKM2 knockdown significantly promotes the apoptin-induced decrease of LDHA protein expression level. These results indicate that apoptin significantly reduces PKM2 protein expression level and inhibits its activity in A549 cells.

The expression of several key glycolysis enzymes, including glucose transporter1 (GLUT-1), hexokinases (HK1and HK2), pyruvate kinase (PKM1 and PKM2) and lactate dehydrogenase (LDHA) was detected by Western blot. The results showed that the expression levels of GLUT-1, HK1, HK2, PKM2, PKM1, and LDHA were significantly decreased in Ad-apoptin treated A549 cells (Fig. **2D**). A similar result was obtained in PKM2-knockdown-cells-where we observed an increased Ad-apoptin-induced downregulation of these proteins. These results showed that Ad-apoptin treatment and PKM2 knockdown inhibit the aerobic glycolysis of A549 cells and that PKM2 knockdown enhances the inhibitory effect of Ad-apoptin on aerobic glycolysis.

To further confirm the relationship between PKM2 and the inhibitory effect of apoptin on glycolysis of cancer cells, PKM2 was transiently overexpressed in A549 cells using plasmid a pcDNA3.1-3xflag-c. The results showed that OE-PKM2 (PKM2 overexpression) significantly increased glucose consumption and lactate production in A549 cells. Ad-apoptin-induced glucose consumption reductions and lactate production were also significantly inhibited (Figs. **2E** and **2F**). The immunofluorescence assay showed that OE-PKM2 inhibits Ad-apoptin-induced downregulation of PKM2 and LDHA in A549 cells (Fig. **2G**). Moreover, we found that OE-PKM2 significantly increases the expression of glycolytic enzymes and reverses the Ad-apoptin-induced decrease of PKM2, GLUT-1, HK1, HK2, PKM1, and LDHA protein expression levels in A549 cells (Fig. **2H**). These results suggest that OE-PKM2 reverses the effect of Ad-apoptin.

### 3.3 Effects of Apoptin and PKM2 Knockdown on the Proliferation, Apoptosis, and Autophagy of A549 Cells

**Fig. (3):**
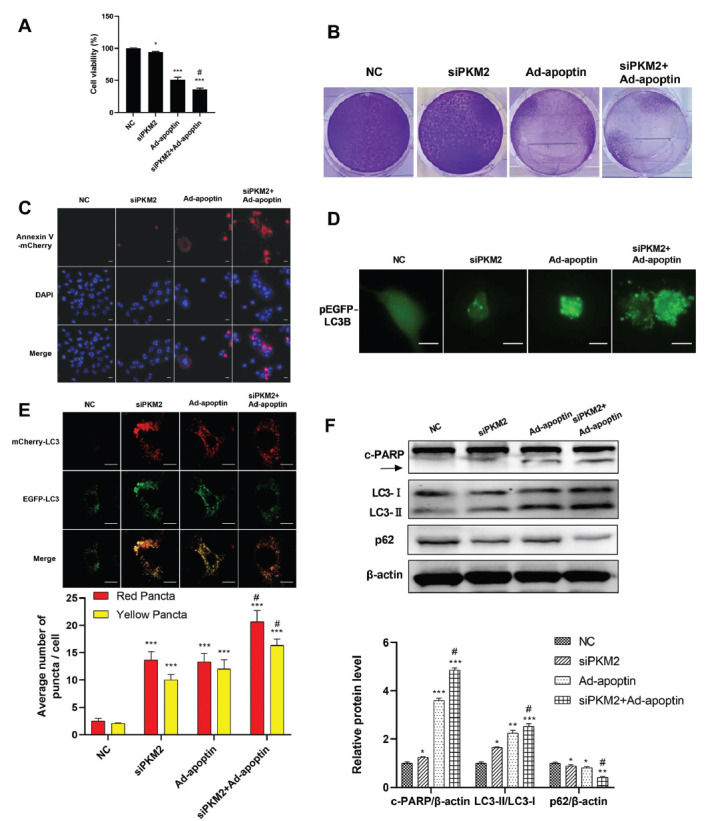
Effects of Ad-apoptin and PKM2 knockdown on proliferation, apoptosis, and autophagy of A549 cells. (**A** and **B**) CCK8 assay (**A**) and crystal violet staining (**B**) were used to detect the proliferation of A549 cells in NC group and treatment group at 36 h. (**C**) Apoptosis was assessed by Annexin V-mCherry staining assay. The cells’ nucleus was stained with DAPI (blue), the cell membranes of apoptotic cells were shown in red fluorescence. (**D**) A549 cells were transfected with pEGFP-LC3B for 36 h and then infected with Ad-apoptin (200 MOI) for 36 h. EGFP signals were detected by fluorescence microscopy (magnification: x400). Bar = 25μm. (**E**) A549 cells were transfected with the mRFP-EGFP-LC3B plasmid. Representative images are shown. Red and yellow puncta were counted and shown as bar diagram. Scale bar = 20 um. (**F**) The levels of PARP, LC3 and P62 were determined by Western blot in A549 cells treated with NC, PKM2 siRNA or Ad-apoptin. *p < 0.05, **p < 0.01 and ***p < 0.001. *vs* the respective NC group; ^#^*p* < 0.05. siPKM2 + Ad-apoptin group or OE-PKM2 + Ad-apoptin group *vs* the respective Ad-apoptin group. n = 3.

To assess the effects of Ad-apoptin and PKM2 on lung cancer cell proliferation, and the CCK-8 assay assessed their survival rate. The results showed that the survival rate of the cells in the siPKM2, Ad-apoptin, and Ad-apoptin + siPKM2 group was significantly inhibited compared with that of the control group. The inhibitory effect in the Ad-apoptin + siPKM2 group was the most significant (Fig. **3A**). In addition, we used crystal violet staining to evaluate the effects of Ad-apoptin and PKM2 knockdown on the ability of A549 single cells to aggregate into active clusters. The colony size and number of A549 cells in the siPKM2 group, Ad-apoptin group, and Ad-apoptin + siPKM2 group were significantly reduced compared with the control group. This effect was a more significant decrease in the Ad-apoptin + siPKM2 group (Fig. **3B**). Moreover, we analyzed the apoptosis of A549 cells using an Annexin v-mcherry staining and the presence of marked red fluorescence staining in the cells of Ad-apoptin and Ad-apoptin + siPKM2 groups (Fig. **3C**), suggesting an induction of apoptosis (red fluorescence) Ad-apoptin and Ad-apoptin + siPKM2 groups. This result was consistent with that of the CCK-8 assay.

To verify the effect of Ad-apoptin and PKM2 knockdown on autophagy in A549 cells, we transfected the pEGFP-LC3B plasmid expressing LC3B and green fluorescent protein into A549 cells and observed the presence of autophagosomes as indicated by the green-fluorescent signals. LC3B positive spots gradually increased in the siPKM2, Ad-apoptin, and the Ad-apoptin + siPKM2 groups; however, no obvious green-fluorescent spots were found in the control group (Fig. **3D**). To investigate whether Ad-apoptin and PKM2 knockdown induce autophagosomes in A549 cells, we transfected A549 cells with the tandem fluorescent-tagged LC3B plasmid mRFP-EGFP-LC3B, which is used to identify autophagosomes (GFP-positive and RFP-positive merge as yellow) and autophagolysosomes (GFP-negative and RFP-positive merge as red) [24]. A549 cells presented an enhanced red and yellow fluorescence in Ad-apoptin + siPKM2 groups (Fig. **3E**), indicating increased autophagosome and autophagolysosome formation.

These results suggest that PKM2 knockdown, Ad-apoptin treatment, and Ad-apoptin + siPKM2 increase the autophagy of A549 cells. This effect was more significant in Ad-apoptin + siPKM2 group. Furthermore, the expression levels of several proteins related to apoptosis and autophagy were analyzed (Fig. **3F**). The results showed that the expression levels of LC3-II and cleaved PARP (c-PARP) in the Ad-apoptin and Ad-apoptin + siPKM2 groups were significantly higher than those in the control group. The increase of c-PARP and LC3-II expression levels was more significant in the Ad-apoptin + siPKM2 group. The expression level of p62 in the Ad- apoptin and Ad-apoptin + siPKM2 groups was significantly lower than that in the control group. The expression level of p62 significantly decreased more in the Ad- apoptin + siPKM2 group. These results suggest that apoptosis and autophagy are increased in the Ad-apoptin and Ad-apoptin + siPKM2 groups. Taken together, these results suggest that PKM2 knockdown and apoptin induce autophagy and apoptosis in A549 cells.

### 3.4 Effects of Apoptin and PKM2 Overexpression on the Proliferation, Apoptosis, and Autophagy of A549 Cells

**Fig. (4):**
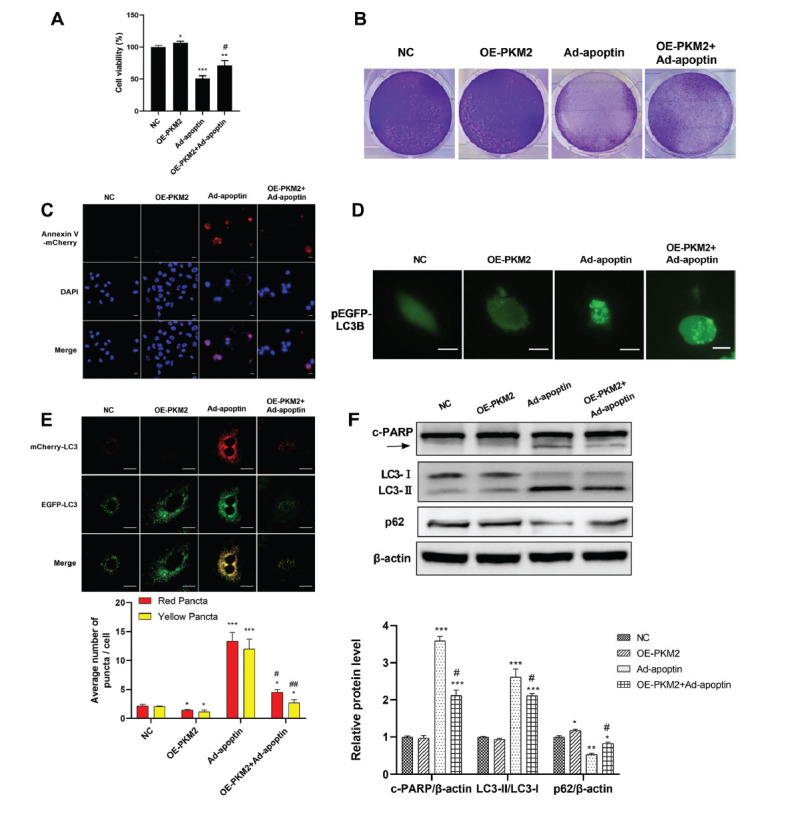
Effects of Ad-apoptin and PKM2 overexpression on proliferation, apoptosis, and autophagy of A549 cells. (**A** and **B**) The proliferation of A549 cells in NC group and treatment group at 36 h was detected by the CCK-8 assay (**A**) and crystal violet staining (**B**). (**C**) Apoptosis was assessed by Annexin V-mCherry staining assay. (**D**) A549 cells were transfected with pEGFP-LC3B for 36 h and then infected with Ad-apoptin (200 MOI) for 36 h. EGFP signals were observed by fluorescence microscopy (magnification: x400). Bar = 25 μm, n = 3. (**E**) A549 cells were transfected with the mRFP-EGFP-LC3B plasmid. Representative images are shown. Red and yellow puncta were counted and shown as bar diagram. Scale bar = 20 um. (**F**) The levels of c-PARP and LC3 were assessed by Western blot from A549 cells treated with NC, PKM2 over-expression or Ad-apoptin. *p < 0.05, **p < 0.01 and ***p < 0.001. *vs* the respective NC group; ^#^*p* < 0.05. siPKM2 + Ad-apoptin group or OE-PKM2 + Ad-apoptin group *vs* the respective Ad-apoptin group. n = 3.

Next, we further investigated the effect of apoptin on lung cancer cells after PKM2 overexpression in A549 cells. For this, the plasmid pcDNA3.1-3xflag-c was used to overexpress PKM2 in A549 cells. The analysis of the cell growth rate was performed using the CCK-8 cell and showed that this rate was significantly higher in the OE-PKM2 group compared with that in the control group. Cell growth in the Ad-apoptin and Ad-apoptin + OE-PKM2 groups was significantly inhibited, while the inhibition of cell growth rate was more significant in the Ad-apoptin group (Fig. **4A**). Crystal violet staining analysis showed that OE-PKM2 significantly promotes the proliferation of A549 cells, while in the Ad-apoptin and Ad-apoptin + OE-PKM2 cell proliferation was significantly inhibited; however, inhibitory effect in the Ad-apoptin group was more pronounced (Fig. **4B**). This result was similar to that of the CCK-8 assay. The Annexin V-mcherry staining showed a marked red fluorescence staining in both Ad-apoptin and Ad-apoptin + OE-PKM2 groups (Fig. **4C**), suggesting an induction of apoptosis in these cells. LC3B positive spots were less observed in that Ad-apoptin + OE-PKM2 group compared with that in the Ad-apoptin group. No obvious green fluorescent spots were found in the control group. The results also showed that OE-PKM2 attenuates the green fluorescence in the presence of Ad-apoptin (Fig. **4D**), further indicating that Ad-apoptin increases autophagy in A549 cells. The final stage of autophagy is the fusion of autophagosomes with lysosomes to form autophagolysosomes. As shown in (Fig. **4E**), compared to the control cells, Ad-apoptin-treated A549 cells showed the presence of a significant overlap between LC3B and lysosomal signals, indicating the formation of autophagolysosomes. Moreover, PKM2 overexpression weakened the accumulation of autophagolysosomes in the presence of Ad-apoptin. Taken together, these results demonstrate that Ad-apoptin increases autophagic flux and the fusion of autophagosomes and lysosomes in A549 cells. In conclusion, PKM2 overexpression attenuates Ad-apoptin-induced apoptosis and autophagy in A549 cells (Figs. **4C - E**).

Western blot analysis showed that the expression levels of c-PARP and LC3-II in Ad-apoptin group and Ad-apoptin + OE-PKM2 group were higher than those in control group. Still, there was little change in the OE-PKM2 group (Fig. **4F**). It is suggested that apoptosis and autophagy increased in the Ad-apoptin and Ad-apoptin + OE-PKM2 groups. Still, there was no change in apoptosis and autophagy in the OE-PKM2 group. PKM2 overexpression partially reversed the effect of Ad-apoptin. In conclusion, these results suggest that PKM2 overexpression provides resistance to Ad-apoptin-induced apoptosis and autophagy of A549 cells.

### 3.5 Apoptin Targets PKM2 and Affects A549 Cells' Metabolism, Autophagy, and Apoptosis through the AMPK/mTOR Pathway

**Fig. (5):**
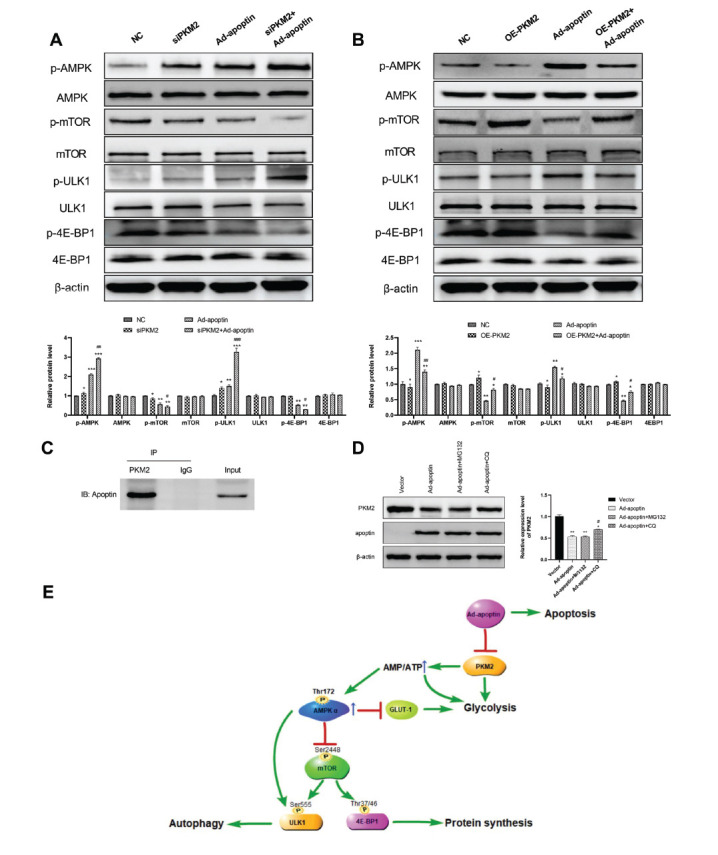
Apoptin targets PKM2 and affects cell metabolism, autophagy, and apoptosis through the AMPK/mTOR pathway. (**A** and **B**) Western blot assessment of the expression levels of p-AMPK, AMPK, p-mTOR, mTOR, p-ULK1, ULK1, p-4E-BP1, and 4E-BP1 in A549 treated with NC, PKM2 siRNA, PKM2 over-expression or Ad-apoptin group. (**C**) The interaction of Apoptin and PKM2 was determined by co-immunoprecipitation (Co-IP) in A549 cells. Apoptin interacts with endogenous PKM2 proteins. (**D**) PKM2 protein expression levels in A549 cells after addition of Ad-apoptin and incubation with MG132 (5 μM), and CQ (20 μM) for 6 hours. (**E**) Ad-apoptin targets PKM2 and affects A549 cells metabolism, proliferation, autophagy, and apoptosis through the AMPK/mTOR pathway. All measurements were performed in triplicate. *p < 0.05, **p < 0.01 and ***p < 0.001. *vs* the respective NC group; ^#^*p* < 0.05 and ^##^*p <* 0.01. PKM2 knockdown or PKM2 over-expression *vs* the respective Ad-apoptin group.

To further explore the molecular mechanism by which apoptin regulates glycolysis in A549 cells, we investigated the expression of some signaling molecules that are involved in cell energy metabolism. AMP-activated protein kinase (AMPK) and serine/threonine protein kinase mTOR are highly sensitive kinases to cell energy status. AMPK is a “metabolic regulator” that helps maintain energy homeostasis in cells. AMPK is frequently downregulated in cancer cells, and its activation often leads to the inhibition of mTOR [25]. ULK1 is a protein necessary for the formation of autophagic vesicles and is regulated by mTOR. 4E-BP1 is an important molecule downstream of the mTOR signaling pathway that mainly regulates protein translation [26]. As shown in After Ad-apoptin treatment or PKM2 knockdown, the level of p-AMPK and p-ULK1 in A549 cells was significantly increased, while the level of p-mTOR and p-4E-BP1 was significantly decreased (Fig. **5A**). PKM2 knockdown further promoted the effect of Ad-apoptin, and its overexpression inhibited the expression of p-AMPK and p-ULK1 and increased the expression of p-mTOR and p-4E-BP1 (Fig. **5B**). Its overexpression also partially reversed Ad-apoptin-induced downregulation of p-mTOR and p-4E-BP1 and upregulation of p-AMPK and p-ULK1 in A549 cells (Fig. **5B**). These results suggest that the Ad-apoptin-induced upregulation of p-AMPK and downregulation of p-mTOR or PKM2 knockdown inhibits the aerobic glycolysis and increases autophagy in A549 cells. The results also suggest that these effects can be partially reversed by PKM2 overexpression and shows that the Ad-apoptin regulatory effect on A549 cells, glycolysis and is closely related to PKM2.

To explore a potential interaction between Ad-apoptin and PKM2, we performed an immunoprecipitation assay and found that apoptin was precipitated by PKM2, suggesting that Ad-apoptin interacts with PKM2 (Fig. **5C**). Next, we investigated whether Ad-apoptin regulates the stability of PKM2 protein. Protein degradation mainly occurs *via* two pathways: The proteasome-dependent pathway and the lysosome-dependent pathway. To study Ad-apoptin mediated PKM2 degradation, A549 cells were first treated with Ad-apoptin and with MG132 (proteasome inhibitor) and CQ (lysosomal inhibitor), respectively. It was found that only CQ treatment inhibits the degradation of PKM2 (Fig. **5D**), indicating that Ad-apoptin promotes PKM2 degradation in a lysosomal-dependent manner. Fig. (**5E**) shows the potential molecular mechanisms by which Ad-apoptin affects tumor energy metabolism, autophagy, apoptosis, and the translation of some proteins through PKM2 targeting.

### 3.6 Ad-apoptin Inhibits Tumor Growth *In Vivo*

To establish a xenograft tumor model in nude mice and further study the anti-tumor effect of Ad-apoptin, A549 cells were subcutaneously injected in mice. After tumor formation, mice were randomly divided into a control and Ad-apoptin groups. The control group was injected with PBS, while the Ad-apoptin group was injected with Ad-apoptin.

**Fig. (6):**
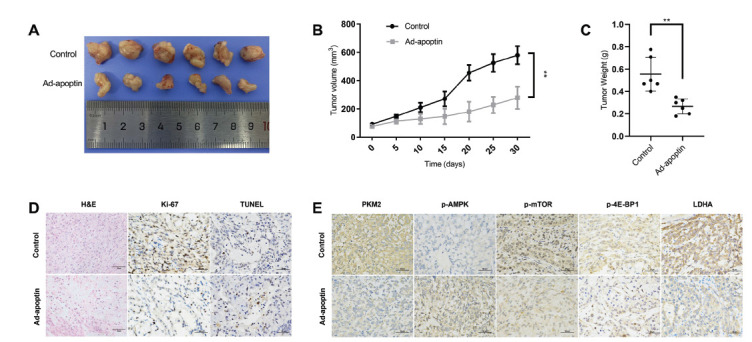
Inhibitory effect of Ad-apoptin on tumor growth *in vivo*. (**A**) Tumors from nude mice treated with Ad-apoptin, and PBS were excised, photographed, and measured. (**B**) The tumor volume curves of the tumors were treated with Ad-apoptin and PBS. (**C**) Tumor weights were measured after the mice had been sacrificed. (**D**) The histological features of the tumors are revealed by H&E. TUNEL staining and Ki-67 staining were used to evaluate the apoptosis or nuclear expression of proliferation markers in animal experimental tumor tissues, respectively. (**E**) Immunohistochemical assessment of PKM2, p-AMPK, p-mTOR, p-4E-BP1, and LDHA expression levels in the tumors. Images are typical of 3 independent assays. Magnification: 400×. Scale bars: 50µm. **p <0.01.

The tumor volume was measured every 5 days for 30 days. On the 30th day, the average tumor volume in the Ad-apoptin group was significantly reduced (278±78.5 mm^3^) compared with that in the control group (579.7±63.9 mm^3^) (*p* < 0.01) (Figs. **6A** and **6B**). In addition, the average tumor weight was significantly lower (0.15±0.06 g) in the Ad-apoptin group compared with that of the control group (0.55±0.27 g) (*p* < 0.01) (Fig. **6C**).

The results of immunohistochemistry showed that the proliferation of tumor cells in the Ad-apoptin group was inhibited and that the cells had nucleolytic and pyknotic fea-tures with more cytoplasmic vacuolation. The Ki-67 assay confirmed that Ad-apoptin significantly inhibits cell proliferation. TUNEL staining showed increased apoptosis in the Ad-apoptin tumors (Fig. **6D**). To further elucidate the mechanism by which Ad-apoptin inhibits tumor growth *in vivo*, we also investigated the expression of related proteins in the tumors’ tissue and found a significant decrease in the expression of PKM2, p-mTOR, p-4E-BP1, and LDHA in Ad-apoptin group compared with that in control group. We also observed an increase in the expression of p-AMPK, which was consistent with the *in vitro* experimental results (Fig. **6E**).

## 4 DISCUSSION

Non-small cell lung cancer (NSCLC) is a common type of lung cancer that has a high mortality rate [27-30]. Therefore, early diagnosis is important but often difficult [31]. To reduce mortality, it is necessary to find a treatment that has low side effects and high efficacy. With the continuous development of molecular biology technology, molecular targeted therapy has become the research focus. In this regard, oncolytic adenoviruses can selectively infect and kill tumor cells and due to their small and easy to modify genome, they can also efficiently express the inserted foreign gene. Thus, oncolytic adenoviruses had become another research hotspot in the development of new antitumor drugs. In this study, a recombinant adenovirus Ad-apoptin expressing apoptin was constructed using the human type 5 adenovirus as a vector. Studies have shown that this vector system can efficiently and stably express the apoptin protein *in vitro* [23]. Previous studies have demonstrated that Ad-apoptin promotes apoptosis and inhibits tumor growth in various types of cancer, including prostate, lung, breast, molecular, and liver cancers *in vitro* and *in vivo*, whereas it has a low cytotoxicity in normal cell lines [19-23, 32-35].

Apoptin is a protein that specifically induces tumor cell apoptosis. Because its effect is neither mediated by p53 nor inhibited by BCL2 overexpression, it is considered as a novel antitumor biological agent [21]. In previous studies, it was found that Ad-apoptin can not only induce tumor cell apoptosis through the intrinsic pathway [36], but also affect autophagy by increasing reactive oxygen species. These effects involved an interactive collaboration between autophagy and apoptosis that promotes cell death of tumor cells [22, 35]. However, as tumor cells have metabolic reprogramming characteristics, the relationship between metabolism and Ad-apoptin has not been elucidated. In this study, human lung cancer A549 cells were infected with the recombinant adenovirus Ad-apoptin expressing human apoptin. The effects of Ad-apoptin on energy metabolism, autophagy, and apoptosis of the tumor cells were studied using a series of experiments. Indeed, we found that apoptin affects the energy metabolism of tumor cells by targeting PKM2 and by affecting the AMPK/mTOR pathway resulting in cell death of tumor cells.

Aerobic glycolysis is usually the main energy source for tumor cells through its supportive role of the tumor invasive and replicative phenotype by providing biosynthetic precursors [37-39]. PKM2 is a key regulator of aerobic glycolysis and the rate-limiting enzyme in the last step of aerobic glycolysis, where it catalyzes the conversion of phosphoenolpyruvate (PEP) and ADP to pyruvate and ATP. It is highly expressed in tumor cells and is closely related to tumor progression [40, 41]. Firstly, we detected the expression of PKM2 in normal lung cells and in several lung cancer cell lines by western blot and qRT-PCR. The results showed that PKM2 expression in lung cancer cells was significantly higher than in normal lung cells. This result is consistent with that from Kaplan-Meier (www.kmplot.com). The high expression of PKM2 is closely associated with a low overall survival in patients with lung cancer (Kaplan-Meier (www.kmplot.com) and according to the data of GEPIA database, the expression level of PKM2 in all tumor samples was higher than that in paired normal tissues. It is suggested that PKM2 may be a useful tumor therapeutic marker. Studies showed that PKM2 can promote aerobic glycolysis by regulating the transcription of genes involved in the aerobic glycolysis pathway, including GLUT-1, LDHA, and HK [24, 42]. We found that after treatment of A549 cells with Ad-apoptin, there is an inhibition of glucose uptake and lactate production and a decrease in cell proliferation rate of A549 cells. Western blot showed that a decrease in the expression levels of PKM2, GLUT-1, HK1, HK2, PKM1, and LDHA decreased in tumor cells and an inhibition of glycolysis and cell proliferation. PARP is the substrate of Caspase-3 and is often used to detect apoptosis. LC3 is a marker protein used to detect autophagy. We found that the expression of c-PARP and LC3-II was increased in Ad-apoptin treated A549 cells, suggesting that apoptin increased apoptosis and autophagy. We also found that PKM2 knockdown in A549 cells led to similar results as those obtained following Ad-apoptin treatment. Moreover, PKM2 knockdown promoted the effect of Ad-apoptin on A549 cells. PKM2 overexpression in A549 cells partially reversed the effect of Ad-apoptin.

Immunoprecipitation is a molecular tool to identify the interaction with specific proteins by binding the target protein specific antibody with the protein A/G affinity beads. Our immunoprecipitation assay showed that apoptin was precipitated by a specific PKM2 antibody of PKM2 indicating that apoptin interacts with PKM2.

We also observed that after Ad-apoptin treatment or PKM2 knockdown in A549 cells, the protein levels of p-AMPK and p-ULK1significantly increased, and the protein levels of p-mTOR and p-4E-BP1 significantly decreased. These results indicate that apoptin increases autophagy and decreases protein synthesis.

According to the above results, apoptin expression in tumor cells results in its interaction with PKM2, leading to PKM2 degradation through the lysosomal dependent pathway, which reduces aerobic glycolysis. We also observed a decrease in tumor cells’ energy production suggesting an effect of apoptin on the cellular energy sensor AMPK. The activation of AMPK has been shown to decrease mTOR phosphorylation which affects its direct downstream targets, such as p-ULK1 and p-4E-BP1, that play key roles in autophagy and protein synthesis [43-45]. Meanwhile, p-AMPK can directly activate p-ULK1 and increase autophagy. The decrease of p-4E-BP1 expression leads to the inhibition of protein synthesis, which may affect the proliferation of tumor cells (Fig. **5D**). Autophagy is a survival mechanism with a double-edged sword. On the one hand, autophagy maintains the normal function of cells, and on the other hand, it leads to cell death (the second programmed death) when it is excessively activated. *In vivo*, we found that the expression levels of PKM2, p-mTOR, p-4E-BP1, GLUT-1, and LDHA decreased, while the expression of p-AMPK increased in nude mice xenografts treated with Ad-apoptin. These results were consistent with the *in vitro* results. It was further confirmed that apoptin inhibits tumor growth and extends the survival time of tumor bearing mice by affecting glycolysis, enhancing autophagy, and inducing apoptosis.

## CONCLUSION

In conclusion, apoptin significantly inhibits the growth of human lung cancer cells. In addition to inducing tumor cell apoptosis through the intrinsic pathway, apoptin increases autophagy and apoptosis by inhibiting aerobic glycolysis. These mechanisms work together to inhibit the growth of tumor cells and induce cell death of tumor cells. This study confirmed the role of apoptin in glycolysis in lung cancer A549 cells. It specifically showed that apoptin targets PKM2 inhibits glycolysis and tumor cell proliferation, and promotes autophagy and apoptosis by regulating the PKM2/AMPK/ mTOR pathway.

## AUTHORS’ CONTRIBUTIONS

Gaojie Song contributed to conceptualization and methodology. Gaojie Song, Yiquan Li and Jinbo Fang contributed to data curation, Writing-Original draft preparation. Chao Shang, Yilong Zhu, Zhiru Xiu, Yaru Li, Xia Yang, Chenchen Ge, and Jicheng Han contributed to visualization, and investigation. Ningyi Jin and Xiao Li supervised the study. Yiquan Li contributed to software, and Validation. Gaojie Song and Jinbo Fang contributed to writing- Reviewing and Editing. Yiquan Li and Jinbo Fang contributed to Funding acquisition.

## Data Availability

The datasets generated during and/or analyzed during the current study are available from the corresponding author (Dr. Yiquan Li) upon reasonable request.

## LIST OF ABBREVIATIONS

PKM2: Pyruvate Kinase M2
NSCLC: Non-Small Cell Lung Cancer
PEP: Phosphoenolpyruvate
IACUC: Institutional Animal Care and Use Committee
GLUT-1: Glucose Transporter 1
HK1and HK2: Hexokinases
PKM1 and PKM2: Pyruvate Kinase
LDHA: Lactate Dehydrogenase
